# 4,4′-{[(2,2′-Bi­pyridine)-5,5′-dicarbon­yl]bis­(aza­nedi­yl)}bis­(1-methyl­pyridin-1-ium) bis­[hexa­fluorido­phosphate(V)] acetonitrile disolvate

**DOI:** 10.1107/S2414314625005176

**Published:** 2025-06-12

**Authors:** Fumika Sueyoshi, Ken Sakai

**Affiliations:** a Department of Chemistry, Faculty of Science, Kyushu University, Motooka 744, Nishi-ku, Fukuoka 819-0395, Japan; Purdue University, USA

**Keywords:** crystal structure, redox-active 2,2-bi­pyridine derivative

## Abstract

The new redox-active title compound, C_24_H_22_N_6_O_2_^2+^·2PF_6_^−^·2C_2_H_3_N, a 2,2-bi­pyridine derivative tethered to two *N*-methyl­pyridinium moieties as electron reservoirs, was synthesized and structurally characterized by ^1^H NMR spectroscopy and single-crystal X-ray diffractometry. The asymmetric unit comprises one half of the divalent bpy cation together with a [PF_6_]^−^ anion and a CH_3_CN mol­ecule. The cation is completed by inversion symmetry. The crystal structure features hydrogen-bonding and π–π inter­actions.

## Abstract 2

In the redox-active title compound, a 2,2-bi­pyridine derivative tethered to two *N*-methyl­pyridinium moieties as electron reservoirs. The asymmetric unit comprises one half of the divalent bpy cation together with a [PF_6_]^−^ anion and a CH_3_CN mol­ecule. The crystal structure features hydrogen-bonding and π–π inter­actions.

## Graphical abstract

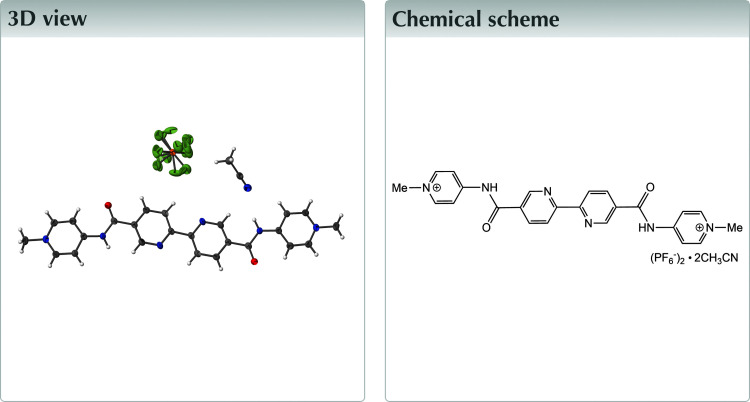

## Structure description

2,2′-Bi­pyridine (bpy) or 1,10-phenanthroline (phen) derivatives have been widely adopted to study various bpy- or phen-ligated transition-metal complexes for a variety of purposes. One of their most remarkable properties lies in the suitably energy.-leveled π*(bpy or phen)-based LUMO, resulting in attractive photochemical and electrochemical properties derived from the redox-active bpy or phen moieties. The [Ru(bpy or phen)_3_]^2+^ family is an important example allowing researchers to advance the knowledge of photochemical and photophysical properties of the ^3^MLCT (triplet metal-to-ligand charge transfer) excited states and their applications to photo-induced electron transfer reactions, especially artificial photosynthetic reactions driven by such photosensitizers.

For example, our previous studies on hybrid molecular systems consisting of an [Ru(bpy)_2_(phen)]^2+^ chromophore and an H_2_-evolving Pt(bpy) catalyst demonstrated a rational strategy to finely tune intra­molecular electron-transfer efficiency together with the associated photocatalytic H_2_ evolution performance (Ozawa *et al.*, 2006 ▸; Masaoka *et al.*, 2010 ▸; Suneesh *et al.*, 2014 ▸). Our studies on the homogeneous water or CO_2_ reduction catalysis have highlighted the importance of raising the electron density at the metal *d* orbitals based on such ligand-based reduction. For example, the Ni(bpy)(di­thiol­ene) H_2_ evolution catalyst was shown to significantly accelerate its proton abstraction rate upon bpy-based reduction (Koshiba *et al.*, 2019 ▸). In general, the performance of cobalt porphyrin CO_2_ reduction catalysts was found to largely rely on the porphyrin-based reductions in order to trigger the CO_2_-binding step, which requires a formal two-electron reduction of CO_2_ to afford a CO_2_^2−^-ligated inter­mediate (Call *et al.*, 2019 ▸; Zhang *et al.*, 2019 ▸, 2021 ▸). A more recent study on Rh(Cp*)(bpy) CO_2_ reduction catalysts further evidenced the inevitable role of an Rh(H)(Cp*)(bpy^.−^) inter­mediate in order to gain a great enhancement in its hydricity to transfer a hydride to CO_2_ to yield a formate anion (Lee *et al.*, 2024 ▸).

In addition to the role of ligand-based reductions, we have also investigated the rate enhancement factors by tethering multiple electron reservoir sites to the ligand coordinated to the catalytically active metal. The Pt(bpy)Cl_2_ catalysts tethered to multiple methyl viologen (*i.e.*, *N*,*N*′-dimethyl-4,4′-bipyridinium) pendants were shown to accelerate the overall photocatalytic H_2_ evolution performance (Kitamoto *et al.*, 2014 ▸, 2016 ▸). The cobalt tetra­kis­(*N*-methyl­pyridinium-*n*-yl)porphyrin (*n* = 2,4) CO_2_ reduction catalysts were also found to exhibit high catalytic efficiencies by accumulation of multiple electrons (Zhang *et al.*, 2019 ▸, 2021 ▸).

With the above issues in mind, a new bpy ligand was designed and synthesized to further explore the role of tethered electron reservoir sites. The two methyl­pyridinium moieties covalently bonded at the 5,5′-positions of the 2,2′-bi­pyridine unit in the title solvated salt, **I**, are expected to serve as additional redox-active sites upon coordinating a catalytically active metal, such as platinum, rhodium, *etc*. As previously described for the cobalt tetra­kis­(*N*-methyl­pyridinium-4-yl)porphyrin catalyst, the two pyridinium moieties linked at both ends of **I** are expected to cooperatively accept electrons using the π-conjugated system. Methyl­pyridinium pendants as cationic moieties will also facilitate water solubility of catalysts fabricated from **I**. Indeed, a preliminary study on the single-mol­ecular photocatalytic H_2_ evolution activity of Pt(**I**)Cl_2_ revealed that it exhibits a higher catalytic rate compared to a control having no methyl­pyridinium pendants (*i.e.*, Pt(5,5′-dicarb­oxy-2,2′-bi­pyridine), the details of which will be separately reported elsewhere.

**Figure 1:**
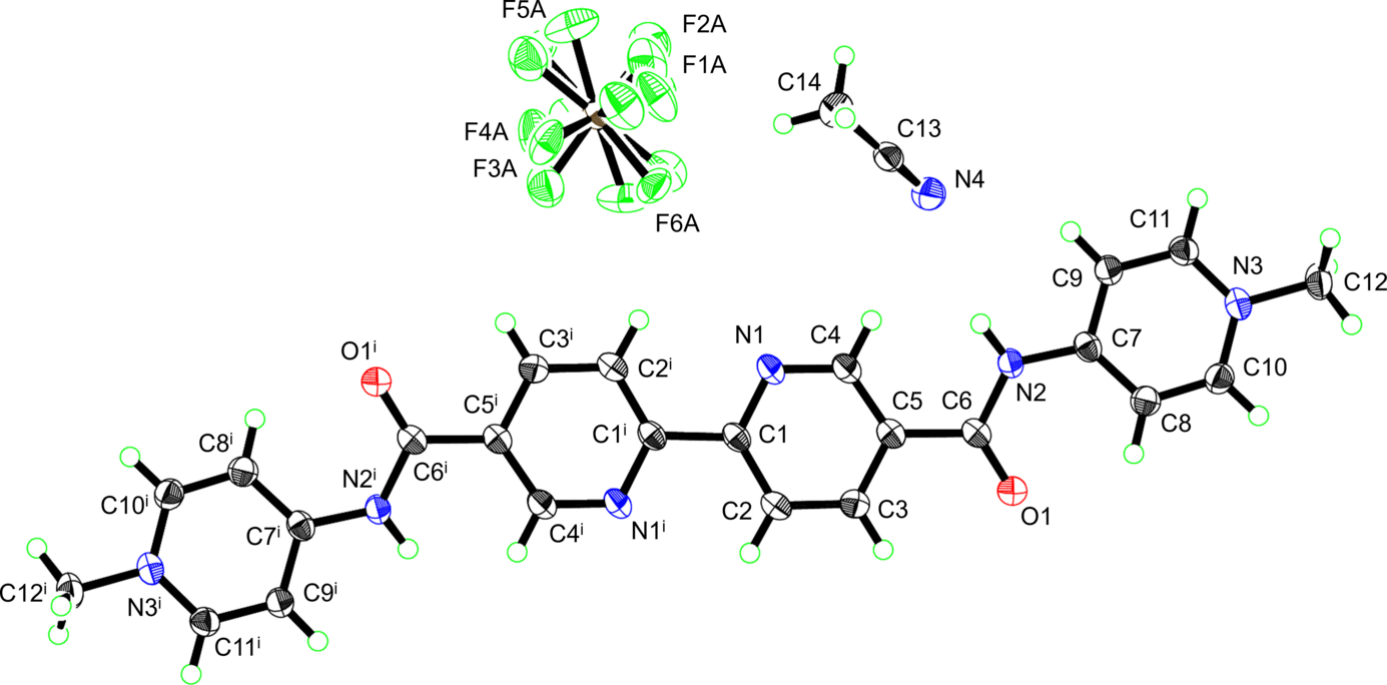
*ORTEP* diagram of **I** with the atom-labeling scheme, where atoms are drawn using the 50% probability displacement ellipsoids for the non-hydrogen atoms. Labels for the minor component of the disordered atoms were omitted for clarity. Symmetry code: (i) −*x* + 1, −*y* + 1, -*z.*

One half of the divalent bpy cation together with a [PF_6_]^−^ anion and a CH_3_CN mol­ecule is found in the asymmetric unit of **I**. The half cationic fragment is bonded to the neighboring fragment through a crystallographic inversion center to give the whole cationic part of the ligand (Fig. 1 ▸), resulting in an almost planar geometry of the central bipyridyl moiety. The central bond distance is expressed by C1—C1^i^ = 1.491 (4) Å [symmetry code: (i) −*x* + 1, −*y* + 1, −*z*] (Table 1 ▸). The two independent pyridyl planes possess a planar geometry with an r.m.s. deviation of 0.011 Å for the N1/C1–C5 plane and 0.006 Å for the N3/C7–C11 plane. Although the dihedral angle between these two pyridyl planes is relatively small [*i.e.*, 1.1 (1)°], they do not form a coplanar geometry because of the twists given by the –C(=O)N– plane connecting the two planes. The dihedral angles of the –C(=O)N– plane with regard to the N1/C1–C5 and N3/C7–C11 planes are 11.2 (2) and −12.3 (2)°, respectively. In other words, the six atoms in one pyridyl plane (*e.g.*, N1/C1–C5) are on average shifted out from the other pyridyl plane (*e.g.*, N3/C7–C11) by 0.33 (2) Å.

**Figure 2:**
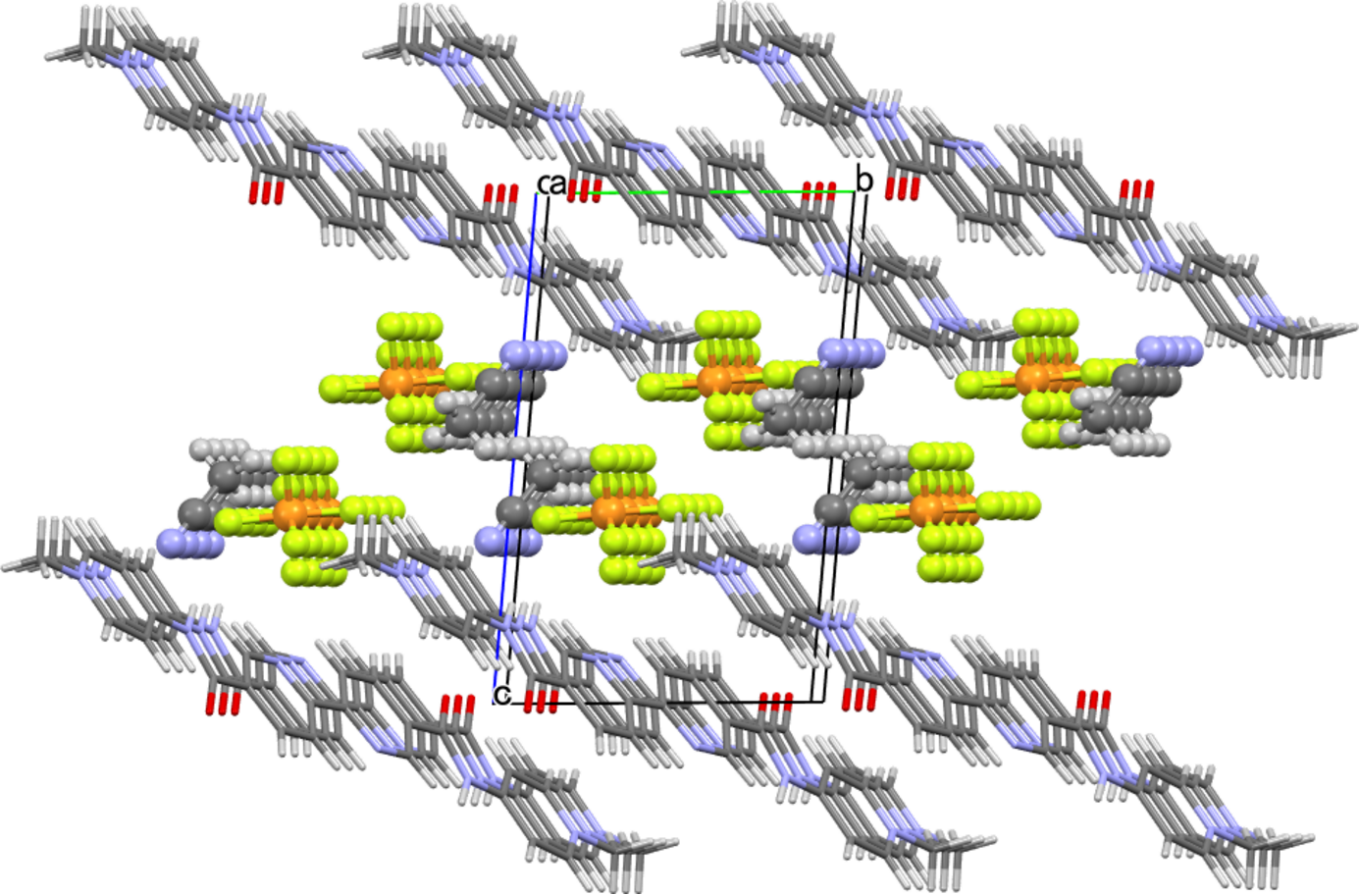
Crystal packing of **I**, viewed along the *a* axis in the range −1 < *a* < 2. Minor disordered PF_6_ moieties are omitted for clarity.

**Figure 3:**
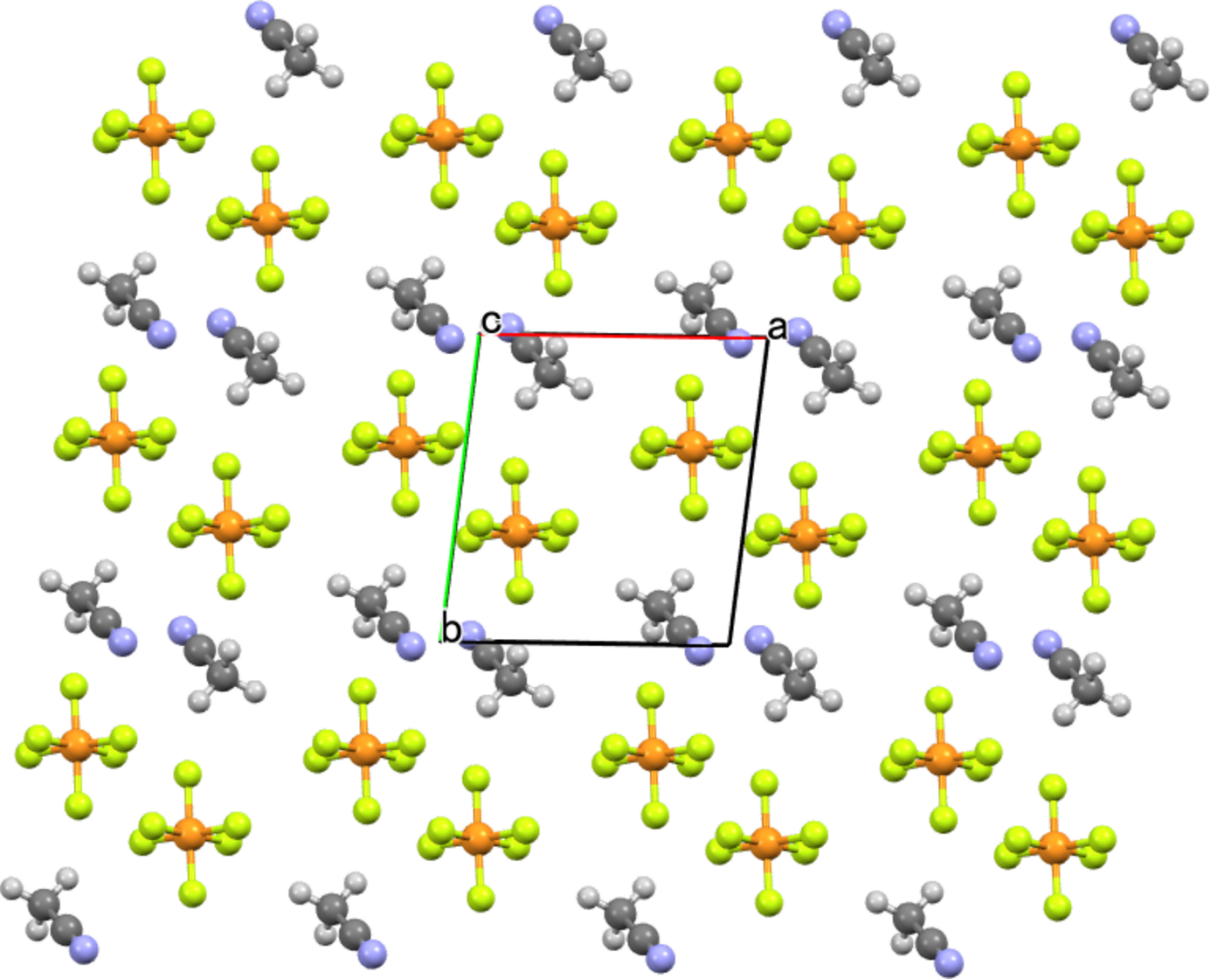
The anionic slab including the aceto­nitrile mol­ecules, viewed along the *c* axis, where the *a* and *b* axes are given in the vertical and horizontal directions. Minor disordered PF_6_ moieties are omitted for clarity.

**Figure 4:**
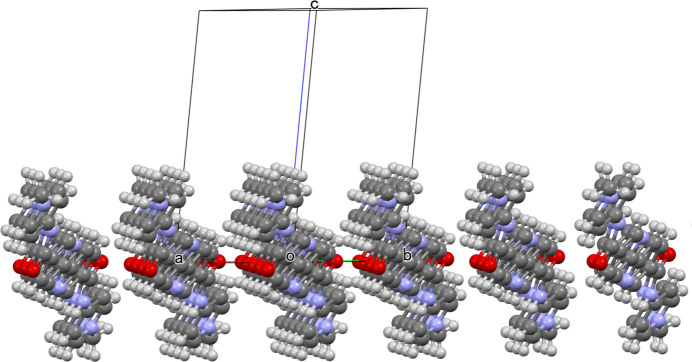
The cationic slab viewed along [110], showing that the 1-D chains are gown by the π–π stacking along this vector with the inter-chain inter­actions formed along [1
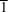
0].

On the other hand, the crystal packing mode is somewhat unique in that the cationic and anionic components respectively form two-dimensional slabs growing parallel to the *ab* plane, electrostatically consolidating the crystal based on the alternate stacks of oppositely charged two-dimensional slabs (Fig. 2 ▸). The aceto­nitrile mol­ecules are involved in the anionic slabs comprised of the [PF_6_]^−^ anions (Fig. 3 ▸). The cationic slabs are consolidated by one-dimensional π–π stacking of the divalent cations together with the hydro­phobic inter­actions formed among the 1-D chains (Fig. 4 ▸). The 1-D chains grow along the [110] direction, while the inter-chain associations perpendicularly grow along the [1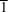 0] direction. The separation between π-stacked planes between the one-dimensionally aligned cations is estimated as 3.45 (2) Å by the results of mean-plane calculations.

**Figure 5:**
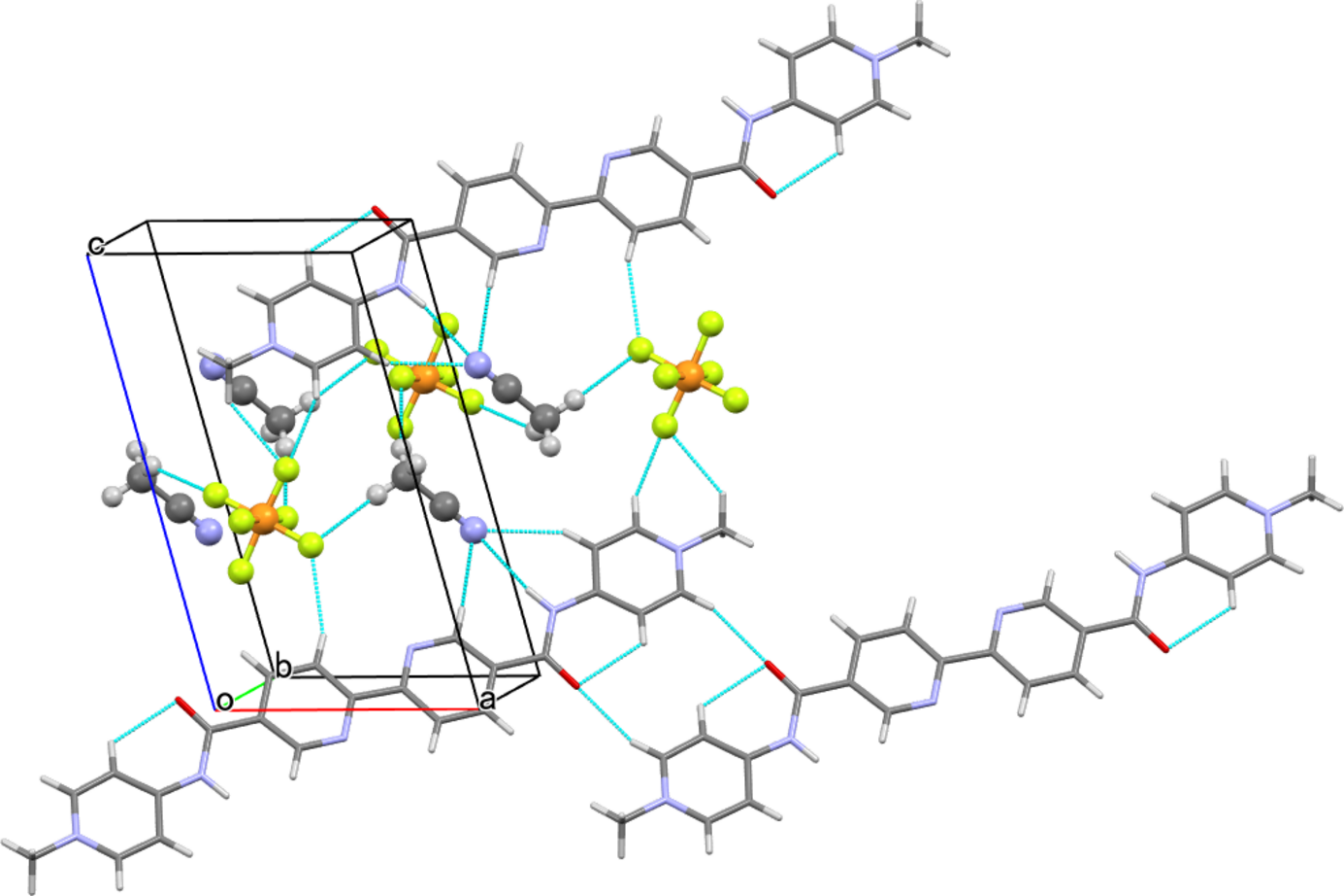
Partial crystal packing of **I**, viewed along the *b* axis, showing hydrogen-bonding inter­actions as dotted lines. Minor disordered PF_6_ moieties are omitted for clarity.

The O atom of the amide is involved in two hydrogen bonds with C8—H8 and C10^iii^—H10^iii^ [symmetry code: (iii) −*x* + 3, −*y* + 2, *z*], exhibiting C⋯O distances of 2.838 (3) and 3.176 (2) Å, respectively (Table 2 ▸, Fig. 5 ▸). The N atom of the aceto­nitrile is involved in three hydrogen bonds with N2—H2*N*, C4—H4, and C9—H9 with N⋯N and C⋯N distances of 3.309 (2), 3.389 (3) and 3.440 (3) Å, respectively (Table 2 ▸, Fig. 5 ▸).

## Synthesis and crystallization

Synthesis of **I**: *N*^5^,*N*^5′^-Di(pyridin-4-yl)-2,2′-bi­pyridine-5,5′-dicarboxamide was prepared by following a literature procedure (Jacobs & Hardie, 2012 ▸). A solution of *N*^5^,*N*^5′^-di(pyridin-4-yl)-2,2′-bi­pyridine-5,5′-dicarboxamide (0.16 g, 0.39 mmol) and iodo­methane (0.19 ml, 3.0 mmol) in aceto­nitrile (70 ml) was refluxed for 24 h. The resulting pale-brown precipitate was collected by filtration, washed with aceto­nitrile (3 × 6 ml), and dried *in vacuo*. The iodide salt of the product was then dissolved in water (*ca* 50 ml) followed by addition of an excess of (NH_4_)[PF_6_] (0.68 g, 4.2 mmol) to give the final product in the form of its hemihydrate as a pale-brown solid, which was collected by filtration, washed with water (3 × 17 ml), and dried *in vacuo* (yield: 0.20 g, 70%). ^1^H NMR (400 MHz, CD_3_CN): δ/p.p.m. = 4.18 (*s*, 6H), 8.28 (*d*, 4H, *J* = 7.0 Hz), 8.45 (*d*, 4H, *J* = 7.4 Hz), 8.48 (*dd*, 2H, *J* = 8.3, 2.4 Hz), 8.70 (*d*, 2H, *J* = 8.3 Hz), 9.27 (*d*, 2H, *J* = 2.4 Hz), 10.01 (*s*, 2H). Analysis calculated for C_24_H_22_N_6_O_2_F_12_P_2_·0.5H_2_O (725.42): C 39.74, H 3.20, N 11.59; found: C 39.63, H 3.01, N 11.51 (%).

Single crystals suitable for the single-crystal X-ray diffractometry were grown by vapor diffusion. The above hemihydrate form of the [PF_6_]^−^ salt dissolved in a minimum amount of aceto­nitrile was sealed within a screw vial under the vapor of diiso­propyl­ether. Upon standing overnight at room temperature, dark-brown plates gradually formed and were collected by filtration. The crystals filtered were relatively stable under ambient conditions and did not lose luster for at least several days.

## Refinement

Crystal data, data collection and structure refinement details are summarized in Table 3 ▸. The [PF_6_]^−^ ion was found to be disordered over three sites with site occupation factors of 0.654 (3), 0.291 (3) and 0.0546 (18). Since there was no disorder problem at the central P atom, only the six F atoms were located over three sites (F1*A*–F6*A*, F1*B*–F6*B*, and F1*C*–F6*C*) and refined anisotropically. The refinement of these 18 disordered F atoms were carried out using SADI and SIMU commands of *SHELXL*. The adjacent F—F distances within the individual site were restrained to be equal using SADI, and the P—F distances were restrained to be common. Moreover, the anisotropic displacement parameters of the disordered atoms were restrained to be similar using SIMU.

## Supplementary Material

Crystal structure: contains datablock(s) I. DOI: 10.1107/S2414314625005176/zl4084sup1.cif

Structure factors: contains datablock(s) I. DOI: 10.1107/S2414314625005176/zl4084Isup2.hkl

CCDC reference: 2457228

Additional supporting information:  crystallographic information; 3D view; checkCIF report

## Figures and Tables

**Table 1:** Selected bond lengths (Å)

O1—C6	1.215 (2)	C1—C1^i^	1.491 (4)
N1—C4	1.335 (3)	C2—C3	1.386 (3)
N1—C1	1.342 (3)	C3—C5	1.391 (3)
N2—C6	1.373 (2)	C4—C5	1.388 (3)
N2—C7	1.387 (2)	C5—C6	1.500 (3)
N3—C11	1.339 (2)	C7—C8	1.392 (3)
N3—C10	1.352 (3)	C7—C9	1.400 (3)
N3—C12	1.477 (3)	C8—C10	1.362 (3)
N4—C13	1.133 (3)	C9—C11	1.366 (3)
C1—C2	1.390 (3)	C13—C14	1.461 (3)

Symmetry code: (i) 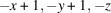.

**Table 2:** Hydrogen-bond geometry (Å, °)

*D*—H⋯*A*	*D*—H	H⋯*A*	*D*⋯*A*	*D*—H⋯*A*
N2—H2*N*⋯F3*C*^ii^	0.89 (3)	2.11 (3)	2.70 (2)	123 (3)
N2—H2*N*⋯N4	0.89 (3)	2.47 (3)	3.309 (2)	157 (3)
C2—H2⋯F6*B*^i^	0.95	2.25	3.037 (7)	139
C4—H4⋯N4	0.95	2.45	3.389 (3)	172
C8—H8⋯O1	0.95	2.26	2.838 (3)	118
C9—H9⋯N4	0.95	2.59	3.440 (3)	150
C10—H10⋯O1^iii^	0.95	2.26	3.176 (2)	162
C11—H11⋯F1*A*^iv^	0.95	2.29	2.981 (4)	129
C11—H11⋯F4*A*^v^	0.95	2.48	3.020 (6)	116
C11—H11⋯F6*A*^v^	0.95	2.48	2.957 (4)	111
C11—H11⋯F1*B*^iv^	0.95	2.46	3.268 (8)	143
C11—H11⋯F1*C*^v^	0.95	2.36	3.21 (2)	150
C12—H12*A*⋯F4*C*^vi^	0.98	2.46	3.19 (2)	131
C12—H12*B*⋯F1*A*^iv^	0.98	2.54	3.348 (4)	139
C12—H12*B*⋯F5*B*^iv^	0.98	2.33	3.272 (7)	160
C14—H14*A*⋯F6*A*	0.98	2.53	3.465 (4)	161
C14—H14*A*⋯F1*B*	0.98	2.50	3.264 (10)	134
C14—H14*A*⋯F6*C*	0.98	2.27	3.24 (2)	172
C14—H14*B*⋯F2*A*^vii^	0.98	2.43	3.397 (5)	171
C14—H14*B*⋯F2*B*^vii^	0.98	2.36	3.334 (13)	176
C14—H14*B*⋯F2*C*^vii^	0.98	2.05	2.941 (14)	151
C14—H14*C*⋯F5*A*^ii^	0.98	2.47	3.176 (4)	129

Symmetry codes: (i) 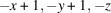; (ii) 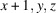; (iii) 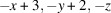; (iv) 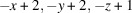; (v) 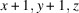; (vi) 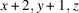; (vii) 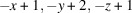.

**Table 3:** Experimental details

Crystal data	
Chemical formula	C_24_H_22_N_6_O_2_^2+^·2PF_6_^−^·2C_2_H_3_N
*M*_r_	798.52
Crystal system, space group	Triclinic, *P* 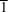
Temperature (K)	100
*a*, *b*, *c* (Å)	7.7661 (2), 8.1535 (3), 13.3262 (5)
α, β, γ (°)	93.392 (3), 102.488 (3), 95.435 (3)
*V* (Å^3^)	817.40 (5)
*Z*	1
Radiation type	Mo *K*α
μ (mm^−1^)	0.25
Crystal size (mm)	0.41 × 0.38 × 0.14

Data collection	
Diffractometer	XtaLAB Synergy, Dualflex, HyPix
Absorption correction	Multi-scan (*CrysAlis PRO*; Rigaku OD, 2024 ▸)
*T*_min_, *T*_max_	0.471, 1.000
No. of measured, independent and observed [*I* > 2σ(*I*)] reflections	10914, 3677, 2834
*R*_int_	0.044

Refinement	
*R*[*F*^2^ > 2σ(*F*^2^)], *wR*(*F*^2^), *S*	0.048, 0.123, 1.09
No. of reflections	3677
No. of parameters	351
No. of restraints	1144
H-atom treatment	H atoms treated by a mixture of independent and constrained refinement
Δρ_max_, Δρ_min_ (e Å^−3^)	0.37, −0.35

Computer programs: *CrysAlis PRO* (Rigaku OD, 2024 ▸), *SHELXT2018/2* (Sheldrick, 2015*a* ▸), *KENX* (Sakai, 2025 ▸), *SHELXL2019/2* (Sheldrick, 2015*b* ▸), *KENX* (Sakai, 2025 ▸), *ORTEPII* (Johnson, 1976 ▸), *TEXSAN* (Molecular Structure Corporation, 2001 ▸) and *Mercury* (Macrae *et al.*, 2020 ▸).

## full crystallographic data

### 4,4'-{[(2,2'-Bipyridine)-5,5'-dicarbonyl]bis(azanediyl)}bis(1-methylpyridin-1-ium) bis[hexafluoridophosphate(V)] acetonitrile disolvate Crystal data

**Table:**

C_24_H_22_N_6_O_2_^2+^·2PF^6^^−^·2C_2_H_3_N	*Z* = 1
*M**_r_* = 798.52	*F*(000) = 406
Triclinic, *P*1	*D*_x_ = 1.622 Mg m^−^^3^
*a* = 7.7661 (2) Å	Mo *K*α radiation, λ = 0.71073 Å
*b* = 8.1535 (3) Å	Cell parameters from 4689 reflections
*c* = 13.3262 (5) Å	θ = 2.7–28.3°
α = 93.392 (3)°	µ = 0.25 mm^−^^1^
β = 102.488 (3)°	*T* = 100 K
γ = 95.435 (3)°	Prism, brown
*V* = 817.40 (5) Å^3^	0.41 × 0.38 × 0.14 mm

### 4,4'-{[(2,2'-Bipyridine)-5,5'-dicarbonyl]bis(azanediyl)}bis(1-methylpyridin-1-ium) bis[hexafluoridophosphate(V)] acetonitrile disolvate Data collection

**Table:**

XtaLAB Synergy, Dualflex, HyPix diffractometer	3677 independent reflections
Radiation source: micro-focus sealed X-ray tube	2834 reflections with *I* > 2σ(*I*)
Mirror monochromator	*R*_int_ = 0.044
Detector resolution: 10.0000 pixels mm^-1^	θ_max_ = 29.6°, θ_min_ = 2.5°
ω scans	*h* = −9→10
Absorption correction: multi-scan (CrysAlisPro; Rigaku OD, 2024)	*k* = −10→10
*T*_min_ = 0.471, *T*_max_ = 1.000	*l* = −14→18
10914 measured reflections	

### 4,4'-{[(2,2'-Bipyridine)-5,5'-dicarbonyl]bis(azanediyl)}bis(1-methylpyridin-1-ium) bis[hexafluoridophosphate(V)] acetonitrile disolvate Refinement

**Table:**

Refinement on *F*^2^	Primary atom site location: dual
Least-squares matrix: full	Secondary atom site location: difference Fourier map
*R*[*F*^2^ > 2σ(*F*^2^)] = 0.048	Hydrogen site location: difference Fourier map
*wR*(*F*^2^) = 0.123	H atoms treated by a mixture of independent and constrained refinement
*S* = 1.09	*w* = 1/[σ^2^(*F*_o_^2^) + (0.0695*P*)^2^ + 0.5331*P*] where *P* = (*F*_o_^2^ + 2*F*_c_^2^)/3
3677 reflections	(Δ/σ)_max_ < 0.001
351 parameters	Δρ_max_ = 0.37 e Å^−^^3^
1144 restraints	Δρ_min_ = −0.35 e Å^−^^3^

### 4,4'-{[(2,2'-Bipyridine)-5,5'-dicarbonyl]bis(azanediyl)}bis(1-methylpyridin-1-ium) bis[hexafluoridophosphate(V)] acetonitrile disolvate Special details

**Table:**

Refinement. All H atoms, except for H2N, were placed in idealized positions (methyl C—H = 0.98 Å and aromatic C—H = 0.95 Å) and included in the refinement in a riding-model approximation, with *U*_iso_(H) = 1.5*U*_eq_(methyl C) and *U*_iso_(H) = 1.2*U*_eq_(aromatic C). Only nitrogen-bound H atom (H2N) was refined isotropically with a fixed isotropic displacement parameter (*U*_iso_(H) = 1.2*U*_eq_(N)).

### 4,4'-{[(2,2'-Bipyridine)-5,5'-dicarbonyl]bis(azanediyl)}bis(1-methylpyridin-1-ium) bis[hexafluoridophosphate(V)] acetonitrile disolvate Fractional atomic coordinates and isotropic or equivalent isotropic displacement parameters (Å^2^)

**Table:**

	*x*	*y*	*z*	*U*_iso_*/*U*_eq_	Occ. (<1)
P1	0.21563 (6)	0.64060 (6)	0.37256 (4)	0.02504 (17)	
F1A	0.3516 (5)	0.6623 (6)	0.4791 (3)	0.0672 (13)	0.654 (3)
F2A	0.2511 (8)	0.8319 (5)	0.3589 (4)	0.0421 (11)	0.654 (3)
F3A	0.0840 (4)	0.6143 (4)	0.2600 (2)	0.0609 (9)	0.654 (3)
F4A	0.1824 (8)	0.4450 (5)	0.3815 (5)	0.0482 (12)	0.654 (3)
F5A	0.0563 (4)	0.6665 (4)	0.4234 (3)	0.0514 (8)	0.654 (3)
F6A	0.3769 (4)	0.6091 (4)	0.3167 (3)	0.0464 (8)	0.654 (3)
F1B	0.3948 (10)	0.6197 (12)	0.4536 (6)	0.057 (2)	0.291 (3)
F2B	0.2841 (17)	0.8322 (11)	0.3722 (11)	0.055 (3)	0.291 (3)
F3B	0.0365 (7)	0.6698 (8)	0.2980 (5)	0.0461 (15)	0.291 (3)
F4B	0.1475 (16)	0.4554 (11)	0.3801 (10)	0.054 (3)	0.291 (3)
F5B	0.1342 (11)	0.6971 (10)	0.4707 (5)	0.0646 (19)	0.291 (3)
F6B	0.3012 (11)	0.5969 (12)	0.2820 (5)	0.065 (2)	0.291 (3)
F1C	0.342 (3)	0.539 (3)	0.4504 (15)	0.063 (5)	0.0546 (18)
F2C	0.285 (3)	0.802 (2)	0.4481 (15)	0.060 (4)	0.0546 (18)
F3C	0.088 (3)	0.741 (3)	0.2974 (15)	0.065 (5)	0.0546 (18)
F4C	0.152 (3)	0.479 (2)	0.2969 (15)	0.055 (4)	0.0546 (18)
F5C	0.064 (2)	0.596 (3)	0.4324 (17)	0.057 (5)	0.0546 (18)
F6C	0.370 (2)	0.684 (3)	0.3140 (17)	0.061 (5)	0.0546 (18)
O1	1.16857 (18)	0.84347 (18)	−0.01190 (11)	0.0331 (4)	
N1	0.6300 (2)	0.6666 (2)	0.08593 (13)	0.0313 (4)	
N2	1.1375 (2)	0.9356 (2)	0.14668 (13)	0.0283 (4)	
N3	1.5859 (2)	1.2694 (2)	0.25358 (13)	0.0276 (4)	
N4	0.8997 (2)	1.0188 (3)	0.31784 (15)	0.0381 (5)	
C1	0.5868 (2)	0.5513 (2)	0.00584 (14)	0.0245 (4)	
C2	0.6944 (3)	0.5290 (2)	−0.06365 (15)	0.0267 (4)	
H2	0.657801	0.448885	−0.120957	0.032*	
C3	0.8559 (3)	0.6259 (2)	−0.04778 (15)	0.0259 (4)	
H3	0.931993	0.613397	−0.094126	0.031*	
C4	0.7863 (3)	0.7586 (3)	0.10044 (16)	0.0311 (5)	
H4	0.817968	0.840171	0.157134	0.037*	
C5	0.9049 (3)	0.7416 (2)	0.03710 (14)	0.0247 (4)	
C6	1.0819 (3)	0.8428 (2)	0.05392 (14)	0.0250 (4)	
C7	1.2920 (3)	1.0428 (2)	0.17970 (15)	0.0258 (4)	
C8	1.4345 (3)	1.0520 (3)	0.13156 (16)	0.0292 (4)	
H8	1.431905	0.979342	0.072716	0.035*	
C9	1.3044 (3)	1.1501 (3)	0.26752 (15)	0.0292 (5)	
H9	1.210315	1.146108	0.302866	0.035*	
C10	1.5775 (3)	1.1657 (3)	0.16923 (16)	0.0305 (5)	
H10	1.673548	1.172358	0.135459	0.037*	
C11	1.4520 (3)	1.2606 (3)	0.30231 (15)	0.0292 (4)	
H11	1.459698	1.332626	0.362209	0.035*	
C12	1.7409 (3)	1.3945 (3)	0.29059 (18)	0.0357 (5)	
H12A	1.838140	1.366523	0.258886	0.054*	
H12B	1.779023	1.396335	0.365756	0.054*	
H12C	1.708218	1.503564	0.271681	0.054*	
C13	0.8309 (3)	0.9568 (3)	0.37464 (17)	0.0325 (5)	
C14	0.7408 (3)	0.8760 (3)	0.44701 (19)	0.0457 (6)	
H14A	0.622944	0.825099	0.410193	0.069*	
H14B	0.728513	0.958103	0.501048	0.069*	
H14C	0.810483	0.790600	0.478350	0.069*	
H2N	1.068 (4)	0.925 (4)	0.192 (2)	0.069*	

### 4,4'-{[(2,2'-Bipyridine)-5,5'-dicarbonyl]bis(azanediyl)}bis(1-methylpyridin-1-ium) bis[hexafluoridophosphate(V)] acetonitrile disolvate Atomic displacement parameters (Å^2^)

**Table:**

	*U*^11^	*U*^22^	*U*^33^	*U*^12^	*U*^13^	*U*^23^
P1	0.0254 (3)	0.0250 (3)	0.0236 (3)	0.0021 (2)	0.0033 (2)	0.0018 (2)
F1A	0.059 (2)	0.094 (4)	0.0334 (16)	−0.018 (2)	−0.0151 (14)	0.0217 (18)
F2A	0.052 (2)	0.0252 (18)	0.053 (2)	0.0034 (13)	0.0191 (19)	0.0070 (14)
F3A	0.0591 (19)	0.071 (2)	0.0362 (15)	−0.0266 (16)	−0.0125 (12)	0.0090 (13)
F4A	0.045 (2)	0.0259 (16)	0.085 (3)	0.0057 (13)	0.036 (2)	0.0163 (16)
F5A	0.0469 (16)	0.0370 (15)	0.080 (2)	0.0065 (13)	0.0356 (15)	0.0024 (15)
F6A	0.0506 (16)	0.0312 (13)	0.066 (2)	−0.0042 (13)	0.0376 (15)	−0.0025 (15)
F1B	0.052 (4)	0.040 (4)	0.062 (5)	0.005 (3)	−0.023 (3)	0.001 (3)
F2B	0.045 (4)	0.037 (5)	0.075 (6)	0.002 (3)	−0.001 (4)	−0.004 (4)
F3B	0.035 (3)	0.033 (3)	0.058 (4)	−0.007 (2)	−0.015 (3)	0.010 (3)
F4B	0.036 (4)	0.043 (5)	0.075 (5)	0.003 (3)	−0.007 (4)	0.013 (4)
F5B	0.088 (5)	0.067 (4)	0.056 (4)	0.028 (4)	0.045 (4)	0.011 (3)
F6B	0.102 (6)	0.064 (4)	0.036 (4)	0.018 (5)	0.030 (4)	−0.002 (3)
F1C	0.062 (8)	0.052 (8)	0.065 (8)	0.007 (8)	−0.005 (8)	0.000 (8)
F2C	0.066 (7)	0.054 (7)	0.059 (7)	−0.001 (7)	0.011 (7)	0.012 (7)
F3C	0.064 (8)	0.055 (8)	0.068 (8)	−0.003 (8)	0.002 (8)	0.003 (8)
F4C	0.054 (7)	0.055 (7)	0.051 (7)	−0.009 (7)	0.008 (7)	0.003 (7)
F5C	0.057 (8)	0.043 (8)	0.069 (8)	−0.002 (8)	0.012 (8)	0.000 (8)
F6C	0.065 (8)	0.054 (8)	0.063 (8)	−0.002 (8)	0.015 (8)	−0.006 (8)
O1	0.0314 (8)	0.0376 (9)	0.0294 (8)	−0.0029 (6)	0.0096 (6)	−0.0037 (6)
N1	0.0289 (9)	0.0338 (10)	0.0265 (9)	−0.0054 (7)	0.0015 (7)	−0.0051 (7)
N2	0.0252 (8)	0.0331 (10)	0.0242 (9)	−0.0057 (7)	0.0047 (7)	−0.0002 (7)
N3	0.0249 (8)	0.0269 (9)	0.0283 (9)	−0.0017 (7)	0.0017 (7)	0.0031 (7)
N4	0.0350 (10)	0.0433 (11)	0.0353 (10)	0.0047 (8)	0.0073 (8)	−0.0016 (9)
C1	0.0269 (10)	0.0223 (10)	0.0207 (9)	0.0016 (8)	−0.0018 (7)	0.0022 (7)
C2	0.0309 (10)	0.0232 (10)	0.0231 (10)	0.0024 (8)	0.0008 (8)	−0.0003 (8)
C3	0.0271 (10)	0.0264 (10)	0.0240 (10)	0.0050 (8)	0.0039 (8)	0.0030 (8)
C4	0.0315 (11)	0.0313 (11)	0.0268 (11)	−0.0031 (9)	0.0031 (8)	−0.0042 (8)
C5	0.0278 (10)	0.0219 (10)	0.0224 (10)	0.0019 (8)	0.0009 (8)	0.0039 (7)
C6	0.0283 (10)	0.0218 (10)	0.0229 (10)	0.0034 (8)	0.0011 (8)	0.0027 (7)
C7	0.0270 (10)	0.0249 (10)	0.0232 (10)	0.0004 (8)	0.0009 (8)	0.0037 (8)
C8	0.0277 (10)	0.0307 (11)	0.0289 (11)	0.0045 (8)	0.0053 (8)	0.0011 (8)
C9	0.0275 (10)	0.0324 (11)	0.0261 (10)	−0.0024 (8)	0.0056 (8)	0.0007 (8)
C10	0.0263 (10)	0.0346 (12)	0.0310 (11)	0.0034 (9)	0.0071 (8)	0.0028 (9)
C11	0.0310 (10)	0.0317 (11)	0.0237 (10)	−0.0005 (9)	0.0051 (8)	0.0017 (8)
C12	0.0295 (11)	0.0345 (12)	0.0395 (12)	−0.0075 (9)	0.0049 (9)	0.0024 (9)
C13	0.0258 (10)	0.0359 (12)	0.0327 (11)	0.0039 (9)	0.0024 (9)	−0.0076 (9)
C14	0.0398 (13)	0.0549 (16)	0.0428 (14)	−0.0025 (11)	0.0144 (11)	−0.0008 (11)

### 4,4'-{[(2,2'-Bipyridine)-5,5'-dicarbonyl]bis(azanediyl)}bis(1-methylpyridin-1-ium) bis[hexafluoridophosphate(V)] acetonitrile disolvate Geometric parameters (Å, º)

**Table:**

O1—C6	1.215 (2)	C7—C9	1.400 (3)
N1—C4	1.335 (3)	C8—C10	1.362 (3)
N1—C1	1.342 (3)	C9—C11	1.366 (3)
N2—C6	1.373 (2)	C13—C14	1.461 (3)
N2—C7	1.387 (2)	P1—F6B	1.538 (7)
N2—H2N	0.89 (3)	P1—F5A	1.559 (3)
N3—C11	1.339 (2)	P1—F1A	1.565 (3)
N3—C10	1.352 (3)	P1—F4B	1.568 (8)
N3—C12	1.477 (3)	P1—F3B	1.573 (5)
N4—C13	1.133 (3)	P1—F3C	1.574 (11)
C1—C2	1.390 (3)	P1—F4C	1.580 (11)
C1—C1^i^	1.491 (4)	P1—F5C	1.585 (11)
C2—C3	1.386 (3)	P1—F2A	1.586 (4)
C3—C5	1.391 (3)	P1—F2C	1.587 (11)
C4—C5	1.388 (3)	P1—F6C	1.590 (11)
C5—C6	1.500 (3)	P1—F1B	1.596 (7)
C7—C8	1.392 (3)		
			
C4—N1—C1	117.76 (17)	C11—C9—C7	119.88 (18)
C6—N2—C7	127.37 (16)	N3—C10—C8	121.53 (18)
C6—N2—H2N	117 (2)	N3—C11—C9	121.25 (18)
C7—N2—H2N	115.5 (19)	N4—C13—C14	179.4 (2)
C11—N3—C10	119.84 (17)	F5A—P1—F1A	92.52 (19)
C11—N3—C12	120.07 (17)	F6B—P1—F4B	92.8 (5)
C10—N3—C12	120.08 (17)	F6B—P1—F3B	92.3 (4)
N1—C1—C2	122.80 (17)	F4B—P1—F3B	91.9 (5)
N1—C1—C1^i^	115.9 (2)	F3C—P1—F4C	90.4 (7)
C2—C1—C1^i^	121.3 (2)	F3C—P1—F5C	90.3 (7)
C3—C2—C1	118.74 (18)	F4C—P1—F5C	91.0 (7)
C2—C3—C5	118.93 (18)	F5A—P1—F2A	92.5 (2)
N1—C4—C5	123.50 (19)	F1A—P1—F2A	90.6 (2)
C4—C5—C3	118.20 (18)	F3C—P1—F2C	90.4 (7)
C4—C5—C6	123.78 (17)	F4C—P1—F2C	178.4 (8)
C3—C5—C6	118.01 (17)	F5C—P1—F2C	90.3 (7)
O1—C6—N2	122.54 (17)	F3C—P1—F6C	90.6 (8)
O1—C6—C5	121.43 (17)	F4C—P1—F6C	89.2 (7)
N2—C6—C5	116.03 (16)	F5C—P1—F6C	179.1 (9)
N2—C7—C8	124.85 (18)	F2C—P1—F6C	89.4 (7)
N2—C7—C9	117.34 (17)	F6B—P1—F1B	90.9 (4)
C8—C7—C9	117.81 (18)	F4B—P1—F1B	89.8 (5)
C10—C8—C7	119.68 (19)	F3B—P1—F1B	176.3 (4)
			
C4—N1—C1—C2	−2.5 (3)	C4—C5—C6—N2	11.2 (3)
C4—N1—C1—C1^i^	178.6 (2)	C3—C5—C6—N2	−169.84 (17)
N1—C1—C2—C3	2.3 (3)	C6—N2—C7—C8	−12.5 (3)
C1^i^—C1—C2—C3	−178.8 (2)	C6—N2—C7—C9	166.98 (19)
C1—C2—C3—C5	0.1 (3)	N2—C7—C8—C10	178.0 (2)
C1—N1—C4—C5	0.3 (3)	C9—C7—C8—C10	−1.5 (3)
N1—C4—C5—C3	2.0 (3)	N2—C7—C9—C11	−178.67 (19)
N1—C4—C5—C6	−179.0 (2)	C8—C7—C9—C11	0.9 (3)
C2—C3—C5—C4	−2.1 (3)	C11—N3—C10—C8	0.4 (3)
C2—C3—C5—C6	178.83 (17)	C12—N3—C10—C8	−178.3 (2)
C7—N2—C6—O1	1.0 (3)	C7—C8—C10—N3	0.8 (3)
C7—N2—C6—C5	−177.87 (18)	C10—N3—C11—C9	−1.1 (3)
C4—C5—C6—O1	−167.70 (19)	C12—N3—C11—C9	177.6 (2)
C3—C5—C6—O1	11.3 (3)	C7—C9—C11—N3	0.4 (3)

Symmetry code: (i) −*x*+1, −*y*+1, −*z*.

### 4,4'-{[(2,2'-Bipyridine)-5,5'-dicarbonyl]bis(azanediyl)}bis(1-methylpyridin-1-ium) bis[hexafluoridophosphate(V)] acetonitrile disolvate Hydrogen-bond geometry (Å, º)

**Table:**

*D*—H···*A*	*D*—H	H···*A*	*D*···*A*	*D*—H···*A*
N2—H2*N*···F3*C*^ii^	0.89 (3)	2.11 (3)	2.70 (2)	123 (3)
N2—H2*N*···N4	0.89 (3)	2.47 (3)	3.309 (2)	157 (3)
C2—H2···F6*B*^i^	0.95	2.25	3.037 (7)	139
C4—H4···N4	0.95	2.45	3.389 (3)	172
C8—H8···O1	0.95	2.26	2.838 (3)	118
C9—H9···N4	0.95	2.59	3.440 (3)	150
C10—H10···O1^iii^	0.95	2.26	3.176 (2)	162
C11—H11···F1*A*^iv^	0.95	2.29	2.981 (4)	129
C11—H11···F4*A*^v^	0.95	2.48	3.020 (6)	116
C11—H11···F6*A*^v^	0.95	2.48	2.957 (4)	111
C11—H11···F1*B*^iv^	0.95	2.46	3.268 (8)	143
C11—H11···F1*C*^v^	0.95	2.36	3.21 (2)	150
C12—H12*A*···F4*C*^vi^	0.98	2.46	3.19 (2)	131
C12—H12*B*···F1*A*^iv^	0.98	2.54	3.348 (4)	139
C12—H12*B*···F5*B*^iv^	0.98	2.33	3.272 (7)	160
C14—H14*A*···F6*A*	0.98	2.53	3.465 (4)	161
C14—H14*A*···F1*B*	0.98	2.50	3.264 (10)	134
C14—H14*A*···F6*C*	0.98	2.27	3.24 (2)	172
C14—H14*B*···F2*A*^vii^	0.98	2.43	3.397 (5)	171
C14—H14*B*···F2*B*^vii^	0.98	2.36	3.334 (13)	176
C14—H14*B*···F2*C*^vii^	0.98	2.05	2.941 (14)	151
C14—H14*C*···F5*A*^ii^	0.98	2.47	3.176 (4)	129

Symmetry codes: (i) −*x*+1, −*y*+1, −*z*; (ii) *x*+1, *y*, *z*; (iii) −*x*+3, −*y*+2, −*z*; (iv) −*x*+2, −*y*+2, −*z*+1; (v) *x*+1, *y*+1, *z*; (vi) *x*+2, *y*+1, *z*; (vii) −*x*+1, −*y*+2, −*z*+1.

## References

[bb1] Call, A., Cibian, M., Yamamoto, K., Nakazono, T., Yamauchi, K. & Sakai, K. (2019). *ACS Catal.* **9**, 4867–4874.

[bb2] Jacobs, T. & Hardie, M. J. (2012). *Chem. A Eur. J.* **18**, 267–276. 10.1002/chem.201101998 22135124

[bb3] Johnson, C. K. (1976). *ORTEPII*. Report ORNL-5138. Oak Ridge National Laboratory, Tennessee, USA.

[bb5] Kitamoto, K. & Sakai, K. (2014). *Angew. Chem. Int. Ed.* **53**, 4618–4622. 10.1002/anie.201311209 24683041

[bb6] Kitamoto, K. & Sakai, K. (2016). *Chem. Commun.* **52**, 1385–1388. 10.1039/c5cc08044d 26616191

[bb7] Koshiba, K., Yamauchi, K. & Sakai, K. (2019). *ChemElectroChem* **6**, 2273–2281.

[bb20] Lee, D., Yamauchi, K. & Sakai, K. (2024). *J. Am. Chem. Soc.* **146**, 31597–31611. 10.1021/jacs.4c09486 39530668

[bb8] Macrae, C. F., Sovago, I., Cottrell, S. J., Galek, P. T. A., McCabe, P., Pidcock, E., Platings, M., Shields, G. P., Stevens, J. S., Towler, M. & Wood, P. A. (2020). *J. Appl. Cryst.* **53**, 226–235. 10.1107/S1600576719014092 PMC6998782 32047413

[bb9] Masaoka, S., Mukawa, Y. & Sakai, K. (2010). *Dalton Trans.* **39**, 5868–5876. 10.1039/c0dt00077a 20502844

[bb10] Molecular Structure Corporation (2001). *TEXSAN*. MSC, The Woodlands, Texas, USA.

[bb11] Ozawa, H., Haga, M. & Sakai, K. (2006). *J. Am. Chem. Soc.* **128**, 4926–4927. 10.1021/ja058087h 16608306

[bb12] Rigaku OD (2024). *CrysAlis PRO* 1.171.43.143a.

[bb13] Sakai, K. (2025). *KENX*. Graphical User Interface for *SHELXL*. Kyushu University, Japan.

[bb14] Sheldrick, G. M. (2015*a*). *Acta Cryst.* A**71**, 3–8.

[bb15] Sheldrick, G. M. (2015*b*). *Acta Cryst.* C**71**, 3–8.

[bb16] Suneesh, C. V., Balan, B., Ozawa, H., Nakamura, Y., Katayama, T., Muramatsu, M., Nagasawa, Y., Miyasaka, H. & Sakai, K. (2014). *Phys. Chem. Chem. Phys.* **16**, 1607–1616. 10.1039/c3cp54630f 24316670

[bb17] Zhang, X., Cibian, M., Call, A., Yamauchi, K. & Sakai, K. (2019). *ACS Catal.* **9**, 11263–11273.

[bb18] Zhang, X., Yamauchi, K. & Sakai, K. (2021). *ACS Catal.* **11**, 10436–10449.

